# Teeth Fibrous

**Published:** 1872-05

**Authors:** James B. Wells

**Affiliations:** Ipswich, Mass.


### Teeth Fibrous.

BY JAMES B. WELLS, D. D. S., IPSWICH, MASS.

The theory of the tubulous structure of the dentine, advanced two hundred years ago by Leuwenhock, and which, until within the past few years, has been the almost universally accepted one, is, we hope, soon to give place to the more rational and better supported theory, that is, of a cellular and fibrous structure.

Retzins finds in the dentine tubes, radiating from the pulp, directed perpendicularly to the surface of the tooth, each tube having three curves. With the aid of a microscope, of 300 diameters, he finds that "the tubes are not simple, but branch off, giving numerous side twigs, which subdivide and occupy the space between the principal tubes." He also says, "that the dentinal part of the tooth is vascular, and under certain circumstances is capable of being injected with red blood, is now well established." This statement is not correct, as it is impossible for blood to be forced into the dentine in any manner, although the tissues might absorb the hematine from the blood, and thus give to the dentine the appearance of being filled with blood. The absurdity of this statement, made by Retzins, has only been excelled by the statement of quite a prominent Professor at a meeting of the Massachusetts Dental Society, during the past year, who said that three blood globules could enter a tubule abreast. Now as a so-called tubule is only one ten-thousandth of an inch in diameter, and a blood globule is almost one three-thousandth, or more than three times as large, the hitch in this case will be readily seen; also, that a man may be well posted in histology, and yet be sadly deficient in mathematics.

Dr. Nasmyth found what he thought to be cellular structure, and that the cells constituted the frame-work in which the osseous matter is deposited, and become the fibres of the dentine.

Later investigations also fail to find any of the hollow tubes so readily seen by Retzins, who must have possessed a vivid imagination, which, although a good thing for a Dickens, will prove to be of little use in histological investigation. But what is a tube? According to Harris, it is a term applied in anatomy to some parts which are hollow. Do we find parts which are hollow in examining a properly prepared section of dentine? No. Such openings can only be found after the organic substances have been completely destroyed. In this case we do not have dentine, but only the earthy remains of it. Professor J. H. McQuillin, of the Philadelphia Dental College, in an article published in volume 7, No. 9, of the Dental Cosmos, says: "The tubuli originate by open mouths, on the walls of the pulp cavity, and proceed in a slightly wavy and radiated manner through every portion of the dentine to its periphery, where they generally terminate, although in some instances extending beyond and penetrating the enamel or cementum, in the latter of which they communicate with the lacunae, through the canaliculi." In volume 13, No. 1, the same author says:

"A section from a recently extracted tooth evidently consists of the hard dentine, and a soft-solid substance, while a section from a tooth which has been removed from the mouth a considerable time, the soft-solid substance drying up, would leave the parts occupied by it hollow or tubular." But does the soft-solid substance dry up and leave the parts hollow or tubular? Not a bit of it. Within the past week, in company with several gentlemen of the Dental profession, I have examined several sections of an incisor tooth, both transverse and longitudinal, which can be proved positively to have been extracted five years, in which not one of the so-called tubuli could be found.

The different sections were first examined with a one-sixth of an inch objective, magnified 400 diameters, then with a one-sixteenth inch, magnified 1,500 diameters. And lastly with a one-fiftieth, immersion, magnified 5 and 10,000 diametors. Small holes were drilled through one transverse section with the point of a needle, these could be distinctly seen under the microscope, but did not resemble a cell in the least.

In preparing one transverse section a hole was accidentally ground through it, which was plain to be seen when placed under the microscope, and not only this, but one of the tubules was seen projecting into this opening quite prominently —a projecting hole. Wonderful! It was, however, beyond a doubt, a cell with nucleus, as its appearance plainly indicated.

To Mr. Tomes is given the honor of first discovering that a number of fibrils projecting from the tubuli, which he regarded as continuations of the nerve fibres of the dental pulp. Dr. Leon S. Beale confirms the statement of Mr. Tomes, and says that "the tubuli are not canals for the passage of nutrient fluids, which transude through the walls and are supposed to pass along the walls to the periphery.

But it is to one of our own time, Professor George B. Harriman, of the Boston Dental College, to whom is due the honor of discovering that fibres radiate from the pulp cavity, and terminate at the junction of the enamel and cementum, and are made up of closely impacted cells with nucleus, around which the calcareous salts are interstitially deposited, thus forming the dentine of the tooth. The same investigator also found minute nerve fibres radiating from the pulp cavity, ramifying the soft-solid substance, and terminating at the junction of the enamel and dentine, which accounts for the extreme sensitiveness almost always found at the line of demarcation between the enamel and dentine. That is the most sensitive part found in excavating outside of the pulp cavity, is a fact so well recognized by all operators that further comment on this part is needless.

We will now speak briefly of cells and their functions.

A cell is a minute body in the tissues devoted to the purposes of nutrition, growth, developement and secretion.

They were first discovered by Professor John Goodsir, and described by him, in the Edinburgh Medical and Surgical Journal, in 1842, as "the typical form of the cell is spherical or oval, but by pressure in growth may assume almost every variety of shape, and their walls become thickened either uniformily or partially."

Histology recognizes the fact that the cell is really the only morphological element in which there is any manifestation of life, and that we cannot transfer the seat of action to any part beyond the cell.

We therefore have in the structure of the dentine these elementary bodies, of which to build up, is eminently characteristic, without the pre-existence of which no living forms arise, and to which the continuance and the maintenance is intimately attached.

Let us then no longer, in the face of such light as this, attribute to hollow tubes with hard walls the function of building up and nourishing organized structure in this part. when all histologists agree that every other part of the body, and not only this, but every living organized substance, is formed and nourished by the cell.

